# Tai Chi on bone mineral density of postmenopausal osteoporosis: A protocol for systematic review and meta-analysis

**DOI:** 10.1097/MD.0000000000021928

**Published:** 2020-09-04

**Authors:** Hai-Yang Wu, Yi-Ru Wang, Guo-Wei Wen, Zhen-Yin Tang, Yi-Qun Yu, Ji-Ren Zhang, Ping Liu, Jun-Hao Wu

**Affiliations:** a Huangpu Branch, Shanghai Ninth People′s Hospital affiliated to Shanghai Jiaotong University, Shanghai, China; b Longhua Hospital affiliated to Shanghai University of Traditional Chinese Medicine, Shanghai, China.

**Keywords:** meta-analysis, osteoporosis, postmenopausal women, review, Tai Chi

## Abstract

**Background:**

Osteoporosis is a clinically common metabolic disease, especially in postmenopausal women. Tai Chi might be beneficial in osteoporosis patients. This study will be performed to examine the effects of Tai Chi on bone mineral density of postmenopausal osteoporosis.

**Methods:**

We will search the electronical databases and hand-searching journals or reference lists. The study screening and data extraction will be carried out by 2 investigators independently. The primary outcome is bone mineral density (lumbar spine, Ward's triangle, trochanter, proximal femur, femoral neck, or total hip). Secondary outcomes are pain score, alkaline phosphatase, osteocalcin, and adverse effects. Review Manager V.5.3 software will be used to compute the data.

**Results:**

The results of the study will provide a reliable evidence to assess the effects of Tai Chi on bone mineral density of postmenopausal osteoporosis.

**Conclusion:**

The conclusion of our systematic review will answer whether Tai Chi is an effective intervention to improve bone mineral density of postmenopausal osteoporosis.

## 1 Introduction

Osteoporosis is the most common bone disease. It is characterized by low bone mass, damage of bone microstructure, increase of bone fragility, and easy occurrence of fractures.^[1]^ In 2001, the National Institutes of Health suggested that reduced bone mass is a major risk factor for osteoporotic fractures.^[2]^ Osteoporosis could occur at any age, but it is more common in postmenopausal women. Postmenopausal osteoporosis (POP) usually occurs within 5 to 10 years after menopause. POP is a systemic disease making the bones brittle and prone to fracture.^[3]^ The lack of estrogen leads to the decrease of bone mass and the change of bone structure in postmenopausal women.^[4]^ Osteoporotic fracture is a major cause of disability and death in elderly patients. Within 1 year of hip fracture, 20% of patients will die of different complications, and about 50% will be disabled with a significantly lower quality of life.^[5]^ The medical treatment and care caused a heavy burden on the family and society. According to a 2015 forecast, medical expenses in China for major osteoporotic fractures (wrist, vertebral, and hip) will reach 132 billion yuan in 2035 and 163 billion yuan in 2050.^[6]^

As a therapeutic exercise, Tai Chi has been practiced for centuries as a martial art in China. At the same time, it has been drawn more and more attention. After being introduced to Europe and America, the viewpoints of Tai Chi shifted and it is nowadays well-known as a kind of exercise or gymnastics.^[7]^ Tai Chi consists of a series of slow and purposeful movements that involve turning, shifting one's weight from one leg to the other one, bending and unbending the legs with various arm movement, which is benefit for balance, flexibility, strength, and health of human beings.^[8]^ Some systematic reviews have shown that Tai Chi exercise may have benefits on bone health in perimenopausal and postmenopausal women, but the evidence is sometimes weak, poor, and inconsistent.^[9,10]^ However, another review believes that the evidence for Tai Chi in the prevention or treatment of osteoporosis is not convincing. More rigorous research seems warranted.^[11]^

Therefore, we intend to use meta-analysis method, search global clinical research about Tai Chi on POP, systematically evaluate the effects of Tai Chi treatment on bone mineral density, and provide more scientific evidence for clinical strategy. In this meta-analysis protocol, we choose bone mineral density (BMD) as the major outcome.

## 2 Methods

We will follow the Cochrane Handbook for Systematic Reviews of Interventions and the Preferred Reporting Items for Systematic Reviews and Meta-Analysis Protocol (PRISMA-P) statement guidelines^[12]^ and illustrate the detailed changes in the full article if needed.

### 2.1 Inclusion criteria

#### 2.1.1 Participants

All patients should be postmenopausal women and also meet the osteoporosis diagnostic criteria established by the Scientific Advisory Board of the European Society for Clinical and Economic Aspects of Osteoporosis (ESCEO) and the Committees of Scientific Advisors and National Societies of the International Osteoporosis Foundation (IOF).^[13]^ There will be no limitations of country, race, and comorbidity. Diagnosis of osteoporosis is as follows:

- Based on the *T*-score for BMD (femoral neck or spine) and a value for BMD 2.5 SD or more below the young female adult mean.
- Low bone mass (osteopenia) should not be considered a disease category but is intended solely for purpose of epidemiological description.

#### 2.1.2 Interventions

Patients in the experimental group should be given with Tai Chi or Tai Chi along with conventional treatment, such as calcium supplement, Vitamin D, alendronate sodium, and so on. The patients in control group should be treated without Tai Chi. It will not be limited to Tai Chi frequency, course, and style (such as Yang Style Tai Chi, Chen Style Tai Chi, Tai Chi pushing hands, or Tai Chi softball exercise, and so on).

#### 2.1.3 Outcomes

- Primary outcomes. BMD (g/cm^2^) of the lumbar spine, Ward's triangle, femoral neck, proximal femur, trochanter, or total hip.
- Secondary outcomes. Pain score, alkaline phosphatase (ALP), osteocalcin (OC) and adverse effects.

#### 2.1.4 Types of studies

All the randomized controlled clinical trials of Tai Chi for POP will be included in this review.

### 2.2 Search methods for identifying the studies

#### 2.2.1 Search sources

We will search the related articles through 3 methods as follows.

- articles included in electronic databases: PubMed, Cochrane Library, Web of Science, China National Knowledge Infrastructure, Chinese Biological and Medical database, and Wanfang Database
- articles only published on paper: relevant journals, conference articles, and dissertations
- unpublished researches to avoid missing grey literature

The search time limit is from the inception to September 2020. Two investigators (HYW and YRW) will independently search for the potential studies according to the pre-established strategy as follows.

(1) Taiji [mh]
(2) (Tai Chi OR Taijiquan OR Tai Ji Quan OR Taiji OR Ji Quan, Tai OR Chi, Tai OR Tai Chi Chuan OR Quan, Tai Ji OR Tai-ji OR T’ai Chi) [tw]
(3) 1 OR 2
(4) osteoporosis [mh]
(5) (Osteoporoses; Osteoporosis, Post-Traumatic; Post-Traumatic Osteoporoses; Osteoporosis, Post Traumatic; Post-Traumatic Osteoporosis; Age-Related Bone Loss; Osteoporosis, Age-Related; Bone Losses, Age-Related; Age-Related Osteoporoses; Osteoporosis, Age Related; Age-Related Bone Losses; Osteoporoses, Age-Related; Age-Related Osteoporosis; Age Related Osteoporosis; Bone Loss, Age Related; Bone Loss, Age-Related; Senile Osteoporosis; Osteoporosis, Involutional; Osteoporosis, Senile; Senile Osteoporoses; Osteoporoses, Senile) [tw]
(6) 4 OR 5
(7) postmenopausal [mh]
(8) (Period, Postmenopausal; Period, Post-menopausal; Post-Menopause; Post-menopausal Period; Post menopausal Period; Postmenopausal Period; Post-Menopauses; Post Menopause) [tw]
(9) 7 OR 8
(10) 3 AND 6 AND 9
(11) randomized controlled trial [pt]
(12) controlled clinical trial [pt]
(13) randomized [tiab]
(14) human trials as topic [mesh: noexp]
(15) randomly [tiab]
(16) trial [ti]
(17) 11 OR 12 OR 13 OR 14 OR 15 OR 16
(18) humans [mh] NOT animals [mh]
(19) 17 and 18
(20) 10 and 19

PubMed search syntax
[mh] denotes a Medical Subject Heading (Mesh) term (‘exploded’)
[tw] denotes text word
[pt] denotes a Publication Type term
[tiab] denotes a word in the title OR Abstract
[sh] denotes a subheading
[mesh: noexp] denotes a Medical Subject Heading (Mesh) term (not ‘exploded’)
[ti] denotes a word in the title.

#### 2.2.2 Search strategy

**Figure 1:**
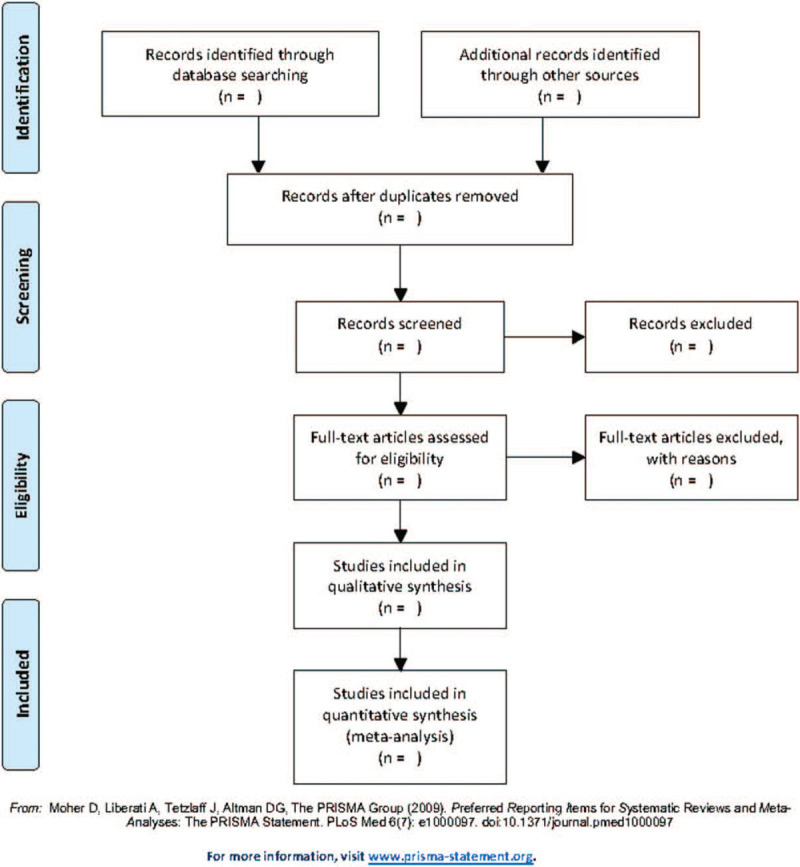
Flow diagram of study selection process.

All the searched articles will be exported to the Endnote software (version 9.3.2; Thomas Reuters, CA) and duplicates will be excluded by software. Two independent investigators (ZYT and GWW) will scan the titles and abstracts of the retrieved studies to finish the initial selection. Next, the full article will be checked if could be included by the same 2 authors. Finally, a third party (JHW) will check whether the selection is same. If there is any difference, JHW will deal with these through discussing and made the final decision. The process of article selection is shown in a flow diagram (Fig. 1).

### 2.3 Data extraction and synthesis

Review Manager (Revman, version 5.3.5; The Nordic Cochrane Centre, Copenhagen, Denmark: http://community.cochrane.org) software will be used to extract literature data by two researchers (YQY and JRZ) independently. A third research author (PL) will check accuracy and consistency of the extracted data. If the data are uncertain, lost or in the form that not extractable, we will contact the authors of the study to request for a confirmation. The data extraction contents are as follows.

- Regular parameters:
  (1) Common information: title, first author name, published time, and contact information
  (2) Postmenopausal women: diagnostic criteria, ethnicity, age, combined disease, and duration of osteoporosis
  (3) Tai Chi intervention: period, frequency and duration of Tai Chi, combined drugs, intervention of the control group, and follow-up
  (4) Trial designs: study type, sample size, baseline balance, randomized and blinding method, completeness and analysis of data

- Meta-analyzed outcomes:
  (1) main outcome: BMD of the lumbar spine, Ward's triangle, trochanter, proximal femur, femoral neck, or total hip)
  (2) secondary outcomes: pain score, ALP and OC, and adverse effects

### 2.4 Risk of bias

Cochrane Collaboration's tool will be used to evaluate the risk of bias by 2 independent reviewers (HYW and YRW). The assessment contains 7 points: sequence generation, allocation concealment, blinding of participants and personnel, blinding of outcome assessors, incomplete outcome data, selective outcome reporting, and other bias. There are 3 levels of each bias according to the Cochrane Handbook for Systematic Reviews of Interventions (Version 5.3): low, unclear, and high level. The disagreements cannot be resolved in this review will search for a third author (JHW) as required. Or else, we will ask the Cochrane Professional Group for a final decision.

### 2.5 Assessment of heterogeneity

We will use Chi-squared test to assess homogeneity of the included studies. If *I*^2^ statistic > 50%, we will consider that there is a significant heterogeneity of the test and use a random effect model; If *I*^2^ statistic < 50%, it means that there is no statistical heterogeneity or heterogeneity is small relatively, therefore, we will use a fixed effect model.

To deal with the significant clinical heterogeneity, we will first check the raw data of the original articles. Then, the sensitivity analysis will be used to find out the reason of heterogeneity. We will also perform subgroup analysis to measure and cope with the heterogeneity due to the following reasons:

(1) Clinical consideration:
  - different age and race
  - different frequency, course, and duration of Tai Chi
(2) Methodology consideration: tests with unclear or high risks of bias

If it is still unable to deal with the heterogeneity, we will choose descriptive analysis.

### 2.6 Evaluation of reporting bias

If an outcome of a meta-analysis contains more than 10 studies, we will adopt funnel plots to determine the risk of reporting bias. Two sides are symmetrical that means there is no obvious publication bias. If the image is unable to make a final conclusion, we will use STATA software (version 12.0; STATA, Texas; https://www.stata.com/) to perform Egger test for quantitative analysis.

### 2.7 Grading the quality of evidence

GRADE profiler software (Version 3.6, GRADEpro GDT, McMaster University, 2015; Evidence Prime, Inc, https://gradepro.org/) will be used to evaluate the quality of evidence. The grades are divided into 4 levels: high, medium, low, and extremely low.

### 2.8 Sensitivity analysis

We will perform the sensitivity analysis to test if possible low-quality studies are included. The detailed method is to remove each included article or some types of articles, then test the *I*^2^ value. This is the main method to assess the robustness and reliability of the synthesized meta-analysis results.

## 3 Discussion

Exercise interventions appear to be effective in preserving postmenopausal women's BMD at the lumbar spine, femoral neck, total hip, and total body.^[14]^ To the best of our knowledge, the newest systematic review and meta-analysis to measure the effect of Tai Chi on BMD of POP is in 2018.^[10]^ Some new clinical studies have been published but not included in the meta-analysis.^[15,16]^ Therefore, we will perform this high-grade analysis to offer a forceful evidence of effects and safety for clinical physician and health policymakers.

However, there might be some potential limitations in this systematic review. For example, different frequencies, courses, and duration of Tai Chi may result in significant heterogeneity and poor methodological quality. We will only include articles published in Chinese or English, which could increase the bias of selection. In addition, different races and ages of the postmenopausal women could also be a heterogeneity risk.

### 3.1 Meta-analysis status

Meta-analysis will start in September 2020 and is expected to finish in March 2021.

## Author contributions

YRW and PL contributed to the conception of the study. The manuscript protocol was drafted by HYW and was revised by JHW. The search strategy was developed by all the authors and will be performed by HYW and YRW. ZYT and GWW will independently screen the potential studies. YQY and JRZ will extract data from the include studies. HYW and YRW will complete the assessment of risk of bias, reporting bias, quality of evidence, data synthesis, and sensitivity analysis. PL and JHW will arbitrate in cases of disagreement and ensure the absence of errors. All authors approved the publication of the protocol.

**Conceptualization:** YRW and PL

**Formal analysis:** HYW and YRW

**Software:** YRW

**Supervision:** JHW and PL

**Writing – original draft:** HYW

**Writing – review & editing:** JHW
